
JOURNAL INFORMATION
==============================
NLM Title Abbreviation: ScientificWorldJournal
Journal ID: ScientificWorldJournal
NLM Journal ID: 101131163
Journal ID: TSWJ

The Scientific World Journal

ISSN: 2356-6140
EISSN: 1537-744X
Publisher: Wiley

ARTICLE INFORMATION
==============================
PMCID: PMC5901311
DOI: 10.1100/tsw.2007.3
Article ID: 413672
Article version: 1
Subjects: Publisher's Note

Publication of a Special Issue: Limb and Fin Regeneration

Redhead Claire
TheScientificWorld, UK
Publication date: 2007
Electronic publication date: 2007 Oct 5
Volume: 7
First page: 1617
Last page: 1617
Copyright: Copyright © 2007 TheScientificWorldJOURNAL
Copyright year: 2007
License: This is an open access article distributed under the Creative Commons Attribution License, which permits unrestricted use, distribution, and reproduction in any medium, provided the original work is properly cited.
License URL: https://creativecommons.org/licenses/by/3.0/

==============================
Publisher's Note
Limb and Fin Regeneration
*TheScientificWorldJOURNAL*, (2007) **7**, 1617
TSW Development & Embryology
ISSN 1537-744X; DOI 10.1100/tsw.2007.3

TheScientificWorldJOURNAL
www.thescientificworld.com

### Publication of a Special Issue: Limb and Fin Regeneration

Claire Redhead
*TheScientificWorld, UK*

**E-mail:** credhead@thescientificworld.com

*Published October 5, 2007*

---

TheScientificWorldJOURNAL and TSW Development & Embryology are delighted to have published the special issue 'Limb and Fin Regeneration', a collection of peer-reviewed papers covering key topics in this important field. The special issue has been organized by Dr. Marie-Andree Akimenko (Ottawa Health Research Institute, Ontario, Canada) and examines regeneration in a range of vertebrate species.

Specific topics covered are:

- Limb regeneration in amphibians: Immunological considerations
- Limb regeneration in Axolotl: Is it superhealing?
- Limb regeneration in *Xenopus laevis* froglet
- Transcriptional profiling of caudal fin regeneration in Zebrafish
- Newt opportunities for understanding the dedifferentiation process
- Comparative experimental analysis of fin lepidotrichia in fishes and several skeletal tissues in vertebrates including humans

The Publisher wishes to express thanks to all of the authors and reviewers, and especially to acknowledge the contribution of Dr. Akimenko in bringing about the publication of these papers.

**This article should be cited as follows:**

Redhead, C. (2007) Publication of a special issue: Limb and Fin Regeneration. *TheScientificWorldJOURNAL:* TSW Development & Embryology **7**, 1617. DOI 10.1100/tsw.2007.3.

©2007 with author.
